# Supplementary material for: Real-time 3D reconstruction from single-photon lidar data using plug-and-play point cloud denoisers
Source: Nat Commun. 2019 Nov 1;10:4984. doi: 10.1038/s41467-019-12943-7 (PMC6825222; doi:10.1038/s41467-019-12943-7)
Supplement: Supplementary file 1 — Supplementary Information [file 41467_2019_12943_MOESM1_ESM.pdf]

Real-time 3D reconstruction from single-photon lidar data using plug-and-play point cloud denoisers

Tachella et al.

Supplementary Information

Supplementary Figures

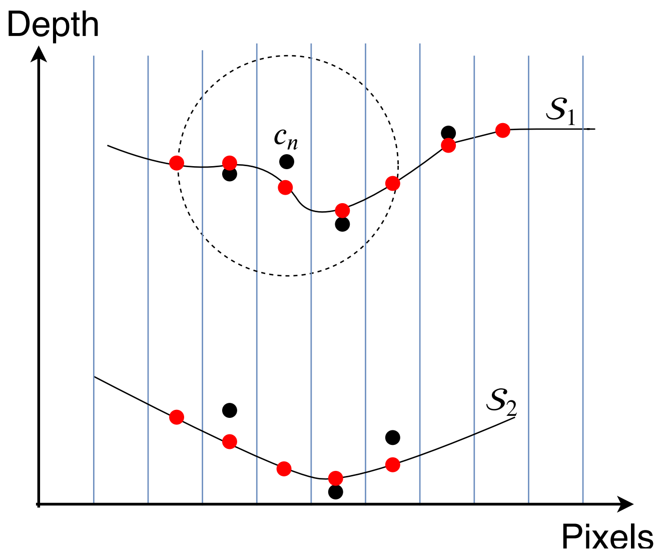

Supplementary Figure 1: Illustration of the APSS denoising step. This example presents two surfaces $\mathcal{S}_1$ and $\mathcal{S}_2$ per pixel. The input and output points are depicted in black and red respectively. The algorithm fits a continuous surface (black line) using local spheres centred at each input point $\mathbf{c}_n$. The fitting is performed using a weighted least squares algorithm, where the weighting kernel is defined by a metric Σ (dashed-line circle). Note that the points in $\mathcal{S}_1$ are not affected by the ones in $\mathcal{S}_2$, as the weighting kernel vanishes at the points in $\mathcal{S}_2$. Thus, the denoiser can process an arbitrary number of surfaces per pixel.

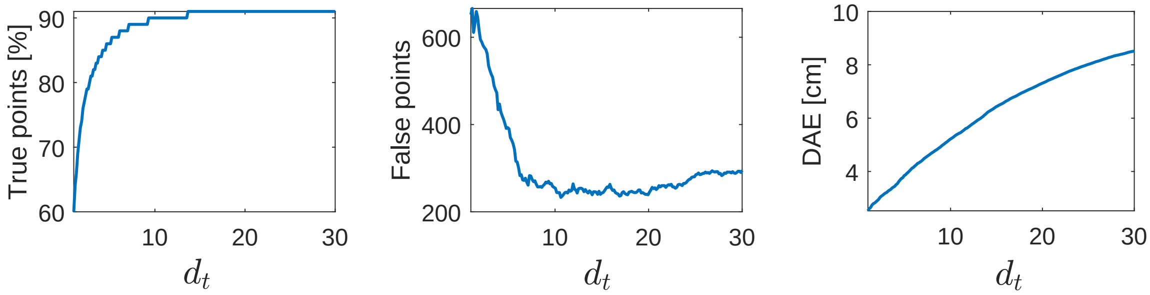

Supplementary Figure 2: Effect of the APSS kernel size d_t. The impact of d_t is shown in terms of true and false detections and mean depth absolute error (DAE) for the "polystyrene head without backplane" dataset. Small values of d_t result in poor reconstructions, as the kernel is too small to correlate neighbouring points, whereas large values oversmooth the depth estimates and may also mix different surfaces. The best choice lies around 8 and 10.

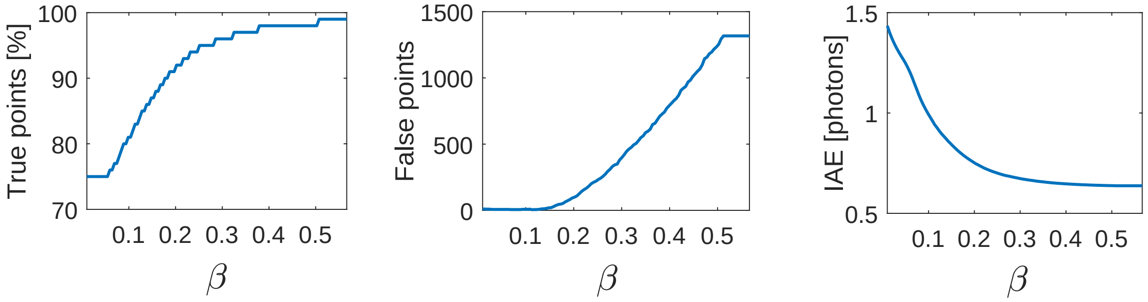

Supplementary Figure 3: Effect of the intensity filtering. The reconstruction quality is shown in terms of true and false detections and mean intensity error (IAE) for the "polystyrene head with backplane" dataset. The reflectivity update depends on the amount of filtering β in the manifold denoising step, which mostly impacts the intensity estimation. Very low values of β mean negligible filtering, finding less points and resulting in a larger intensity error. Large values (close to 1) oversmooth the estimates, generating false detections and also resulting in a larger intensity error (this effect is reduced by the very smooth profile of a polystyrene head). Good values for β generally lie in the interval $[0.1, 0.3]$. Note that this interval might vary depending on the number of pixels of the array.

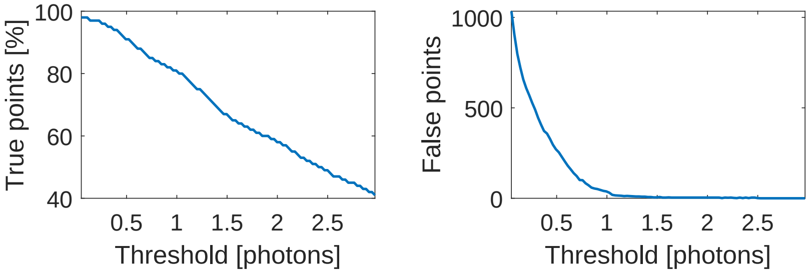

Supplementary Figure 4: Effect of the intensity threshold $r_{\min}$. Number of true and false detections as a function of the intensity threshold for the "polystyrene head with backplane". As we increase the threshold, the number of true detections decreases monotonically. In contrast, the number of false detections increases exponentially as the threshold tends to zero. The best performance is obtained for values between 0.2 and 0.4 photons, coinciding with the reflectivity interval from 5% to 10%. This interval can be used as a guideline for setting $r_{\min}$. The execution time is not affected significantly by the threshold, as the complexity is mostly driven by the (fixed) number of photons.

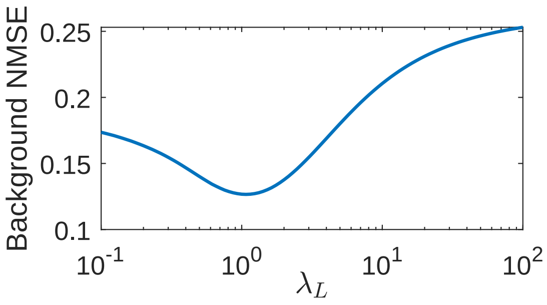

Supplementary Figure 5: Effect of background regularisation λ_1. The background performance (in terms of NMSE) is shown as a function of λ_1 for the "polystyrene head without backplane". The background update depends on the hyperparameter λ_1, which controls the degree of correlation between neighbouring background levels. While low values of λ_1 do not impose sufficient correlation, large values of λ_1 tend to oversmooth the estimates. While the best choices lie in the interval $[0.5, 2]$, the performance is not very sensitive to bad specifications of λ_1.

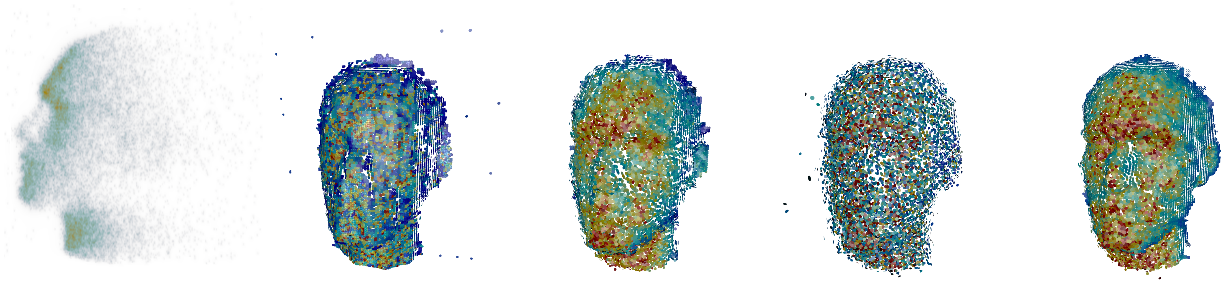

Supplementary Figure 6: Comparison in a target detection setting. Reconstructions achieved by the proposed algorithm and competing methods for the “polystyrene head without backplane” scene. The colour scheme denotes the number of returned photons attributed to each 3D point. The dataset presents at most one surface per pixel. In this case, if a single-surface per pixel algorithm [1] plus a thresholding step is used, the borders of the target are correlated with spurious detections in pixels without surfaces, yielding relatively poor estimates. The target detection algorithm takes into account the presence of pixels without any surfaces, but does not promote any correlation between detected points. Both the proposed method and ManiPoP provide good results, correlating only points belonging to the target.

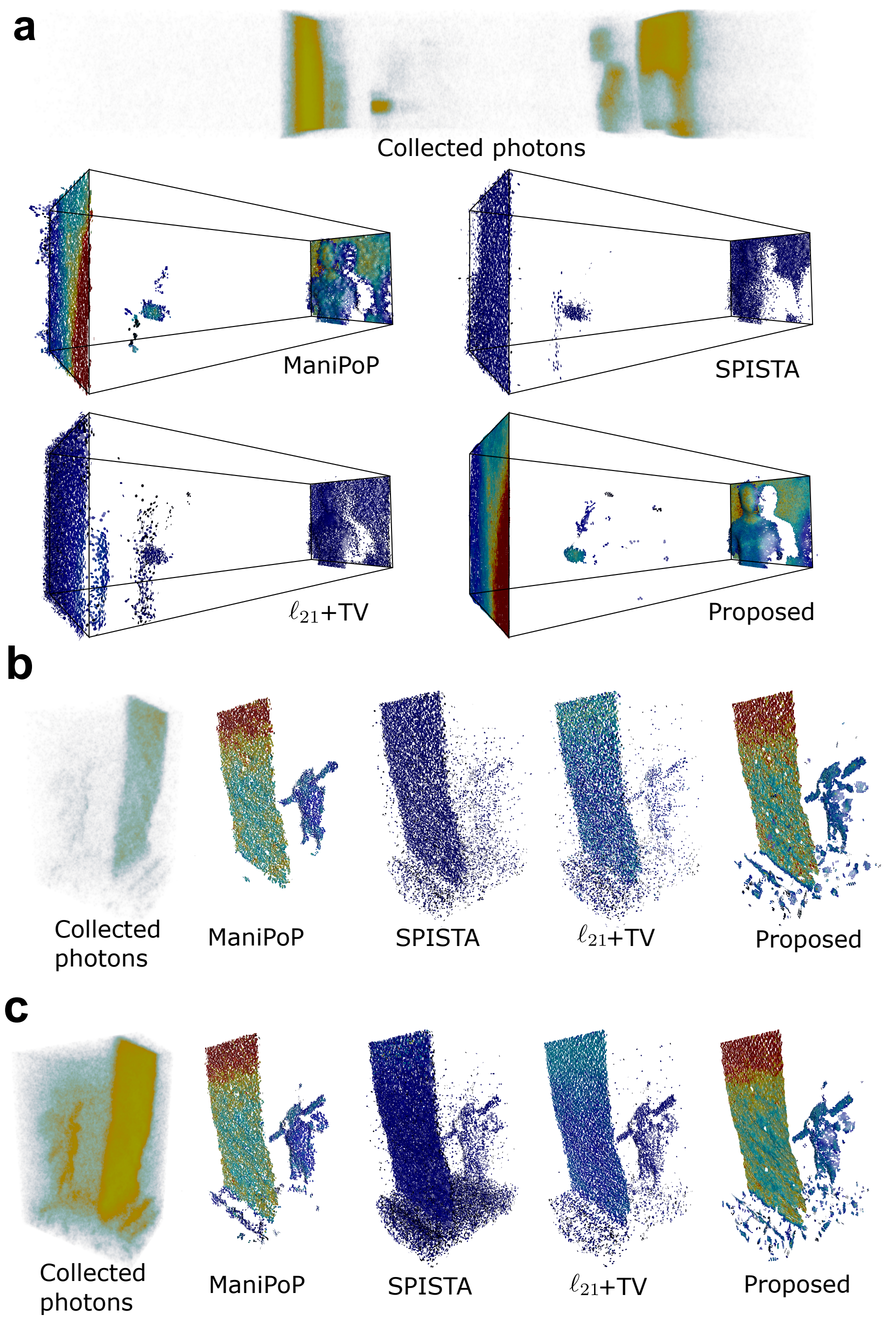

Supplementary Figure 7: Comparison in the presence of multiple surfaces per pixel. Reconstructions achieved by the proposed algorithm and competing methods for (**a**) "mannequin behind scattering object" and "human behind camouflage netting" using acquisition times of (**b**) 0.32 ms and (**c**) 3.2 ms. In this scene, single-depth algorithms, including cross-correlation, cannot be applied, as they would only reconstruct the first object. In these cases, we evaluate SPISTA, ℓ_{21}+TV, ManiPoP and the proposed method, which can handle multiple surfaces. The best results are obtained by ManiPoP and the proposed algorithm. However, ManiPoP requires an execution time many orders of magnitude higher than the novel method.

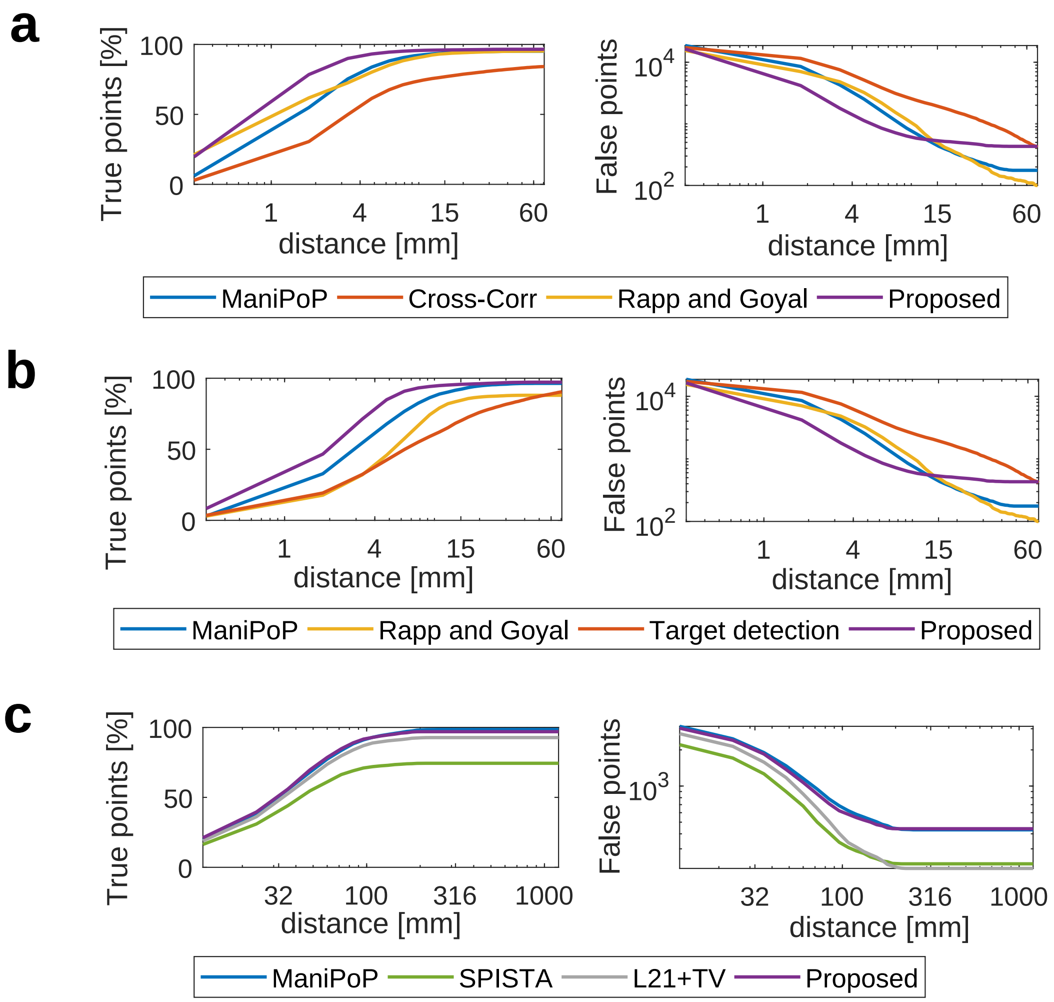

Supplementary Figure 8: Quantitative comparison of reconstruction algorithms. The number of true and false detections are shown for (**a**) "polystyrene head with backplane", (**b**) "polystyrene head without backplane" and (**c**) "mannequin behind scattering object". The number of true and false detections are shown as a function of the maximum admissible distance between a ground truth point and a detected one. The proposed method finds more true points than other state-of-the-art algorithms with less depth error.

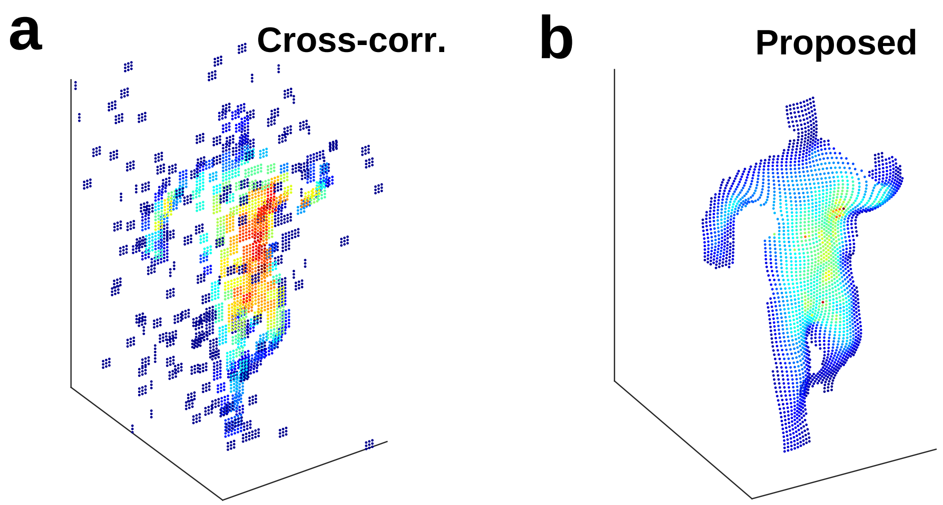

Supplementary Figure 9: Super-resolution using the 32×32 lidar array data. (**a**) Naively upsampled cross-correlation estimate. (**b**) Reconstruction obtained by the proposed method, where the upsampling is formulated in the observation model. The proposed upsampling can bring additional details to the reconstructed objects, improving the estimates of naive upsampling in a post-processing step. The cross-correlation output was upsampled by converting each detection into a 3×3 grid of points at the same depth. While the upsampled cross-correlation has a blocky appearance, the proposed method captures additional details in the contours of the 3D target. Note that these contours are not necessarily aligned with the coarse scale.

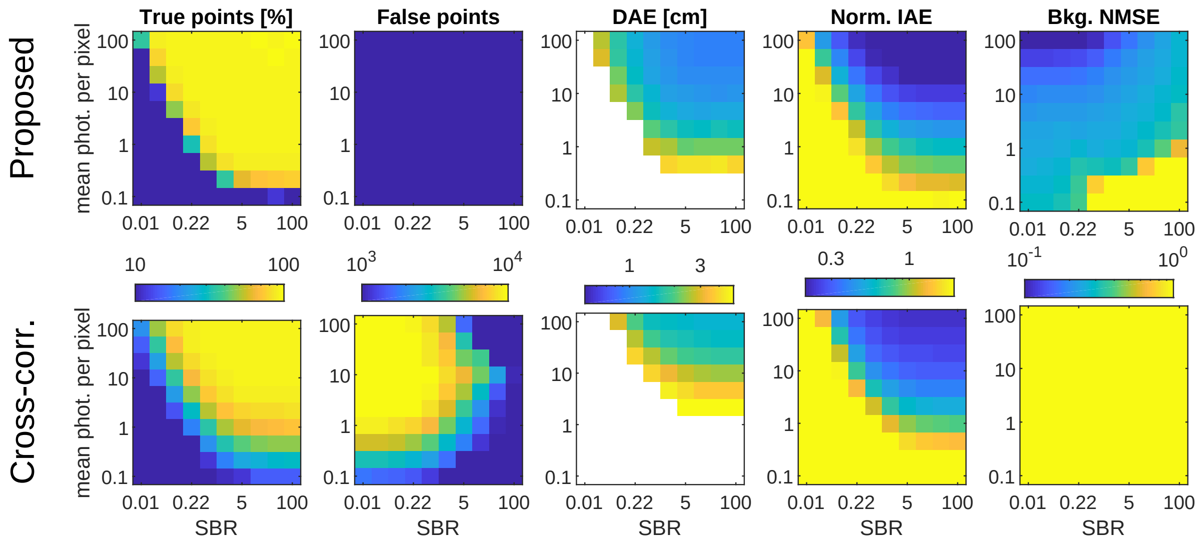

Supplementary Figure 10: Operation boundary conditions. Comparison of the proposed method and cross-correlation with thresholding in a target detection setting for different SBR and mean photons per pixel values. We consider the number of true and false detections, depth absolute error (only computed for true detections and reconstructions with more than 80% of detected points and left blank otherwise), intensity absolute error (normalised by the mean signal photon counts to approximately lie between 0 and 1) and background NMSE. The proposed method performs well in a wider range of conditions, achieving good reconstructions with up to ≈ 0.1 photons per pixel and up to SBR of 0.01 (with 100 photons per pixel or more).

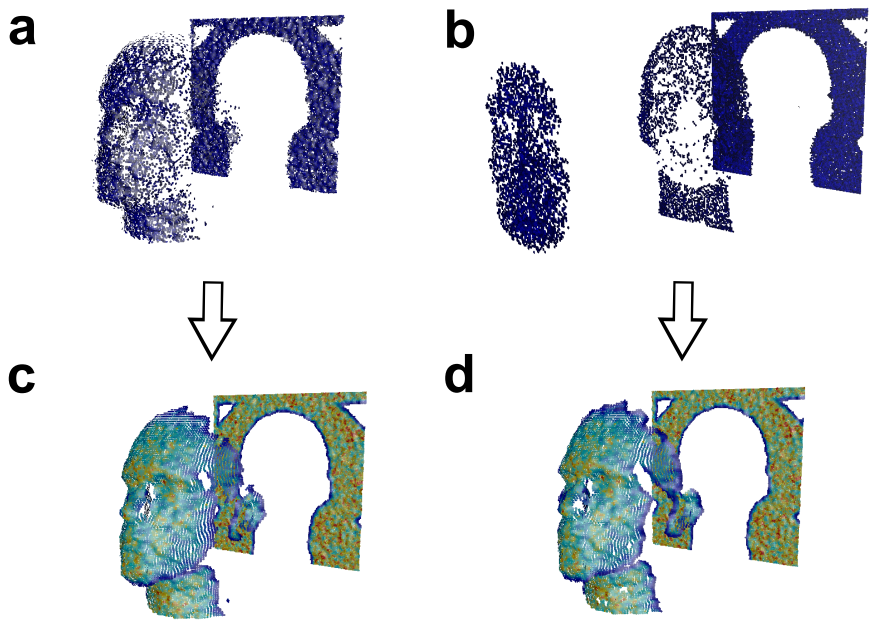

Supplementary Figure 11: Reconstructions from different initialisations. Figures (**a**) and (**b**) show the initialisation of the algorithm when computing the 100% and 0.11% of the total cross-correlation. The reconstructions obtained after running the proposed reconstruction algorithm are shown in (**c**) and (**d**). Despite using different initialisations, the algorithm converges to similar reconstructions.

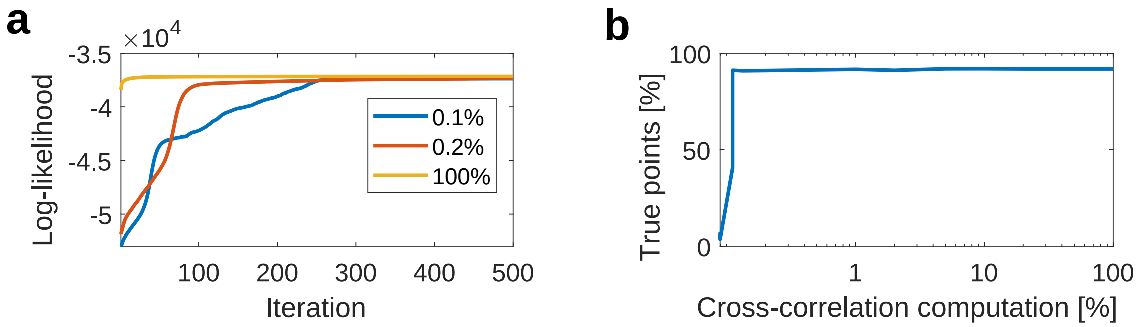

Supplementary Figure 12: Robustness to the initialisation. (**a**) Value of the log-likelihood as a function of the iterations of the proposed algorithm for different initialisations. In all cases, the algorithm converges to a similar maximum of the log-likelihood function. (**b**) Ground truth points found using different initialisations by reducing the computation of the cross-correlation.

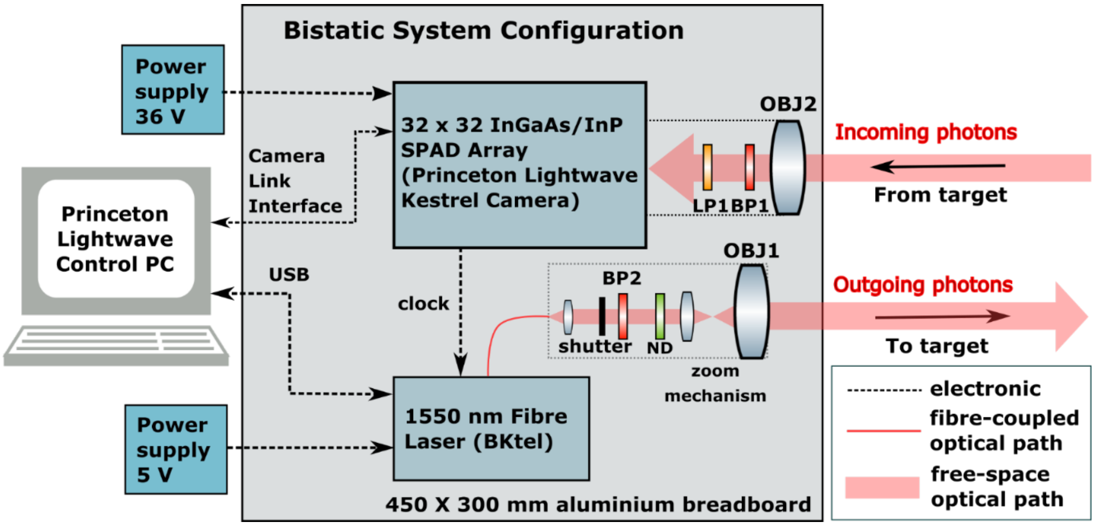

Supplementary Figure 13: Schematic diagram of the lidar system. The key components and configuration of the bistatic imaging system comprise the Princeton Lightwave Kestrel 32×32 InGaAs/InP SPAD detector array and the $\lambda = 1550$ nm fibre pulsed illumination source. Optical components include: objective lenses (OBJ1, OBJ2)); a neutral density filter (ND); a longpass filter (LP1); and bandpass filters (BP1, BP2). Details of these components are given in the Supplementary Methods.

Supplementary Tables

Dataset	Polystyrene head [2] with backplane	Polystyrene head without backplane [3]	Human behind camouflage netting [4]	Mannequin behind scattering object [5]
N_r	141	141	159	99
N_c	141	141	78	99
T	4613	2500	550	4001
Binning resolution	0.3 mm	0.3 mm	1.2 mm	5.6 mm
Mean phot. per pixel	3.37	1.14	44.6 (long. acq.)<br>4.5 (short acq.)	45
SBR	13.62	8.14	2.35	8.57
Stand-off distance	40 m	40 m	230 m	4 m
Applicable algorithms	ManiPoP [3],<br>Rapp and Goyal [1]<br>Cross-corr.	ManiPoP [3]<br>Target detection [6]<br>Rapp and Goyal [1]<br>with thres.	ManiPoP [3]<br>SPISTA [5]<br>ℓ_{21}+TV [7]	ManiPoP [3]<br>SPISTA [5]<br>ℓ_{21}+TV [7]

Supplementary Table 1: Summary of the evaluated raster-scanning lidar datasets. N_r and N_c are the number of vertical and horizontal pixels, T is the number of histogram bins and SBR is the signal-to-background ratio.

	Polystyrene head with backplane	Polystyrene head without backplane	Human behind camouflage netting	Mannequin behind scattering object
Parallel cross-corr	1 ms	1 ms	NA	NA
SPISTA [5]	705 s	3362 s	1279 s (long. acq.)<br>1212 s (short acq.)	2871 s
ℓ_{21}+TV [7]	201 s	187 s	165 s (long. acq.)<br>182 s (short acq.)	202 s
Rapp and Goyal [1]	37 s	44 s	NA	NA
Target detection [6]	12 h	12 h	NA	NA
ManiPoP [3]	201 s	181 s	120 s (long. acq.)<br>102 s (short acq.)	146 s
Proposed method	13 ms	11 ms	27 ms (long. acq.)<br>15 ms (short acq.)	40 ms

Supplementary Table 2: Execution time of the reconstruction algorithms. Some methods do not provide meaningful results in certain scenes. For such cases, the execution time is not available (NA). The proposed method presents a higher computing time than a parallel implementation of the cross-correlation algorithm (which only applies in the presence of single peaks), but outperforms all the other reconstruction algorithms by a factor of about $\approx 10^5$.

Supplementary Note 1: Details of the 3D reconstruction algorithm

The 3D reconstruction task consists of recovering depth $\mathbf{t}$ and intensity $\mathbf{r}$ information from the lidar data $\mathbf{Z} \in \mathbb{Z}_{+}^{N_{\mathrm{r}} \times N_{\mathrm{c}} \times T}$, where the photons recorded in pixel (i, j) and histogram bin t are denoted by $z_{i,j,t}$. However, the (vectorised) background image $\mathbf{b} \in \mathbb{R}_{+}^{N_{\mathrm{r}} N_{\mathrm{c}}}$ should also be estimated from the data, as it is generally a priori unknown and it has a strong impact on the estimation of $\mathbf{t}$ and $\mathbf{r}$. Consequently, we also estimate $\mathbf{b}$ in addition to the depth and intensity profiles. In the single-surface per pixel setting, $\mathbf{t} \in \mathbb{R}^{N_{\mathrm{r}} N_{\mathrm{c}}}$ and $\mathbf{r} \in \mathbb{R}_{+}^{N_{\mathrm{r}} N_{\mathrm{c}}}$ are vectorised images of fixed size, whereas in the multiple-surface per pixel setting $\mathbf{t}$ and $\mathbf{r}$ are sets of $N_{\mathbf{\Phi}}$ points, which is a priori unknown.

Previous 3D reconstruction algorithms

In a fixed dimensional setting (fixed number of points) and assuming one surface (point) per pixel and negligible background levels, the maximum likelihood estimate (MLE) is

$$(\hat{\mathbf{t}}, \hat{\mathbf{r}}) = \underset{\mathbf{t},\mathbf{r}}{\arg\min}\, g\left(\mathbf{t}, \mathbf{r}, \mathbf{b} = \mathbf{0}\right) \tag{1}$$

which corresponds to cross-correlating the data with $\log h_{i,j}(t)$ and finding the delay leading to the maximum correlation for each pixel. When the number of photons per pixel is low, or when the single object per pixel assumption does not hold or when the background levels are not negligible, the MLE does not provide reliable estimates. These estimates can be improved by considering a priori information on the structure of $\mathbf{\Phi}$ and $\mathbf{b}$. One approach, referred to as penalised maximum-likelihood (PML), introduces additive regularisation terms $\rho_{\mathbf{t}}(\mathbf{t})$, $\rho_{\mathbf{r}}(\mathbf{r})$ and $\rho_{\mathbf{b}}(\mathbf{b})$ to enforce more structured solutions, that is

$$(\hat{\mathbf{t}}, \hat{\mathbf{r}}, \hat{\mathbf{b}}) = \underset{\mathbf{t},\mathbf{r},\mathbf{b}}{\arg\min}\, g\left(\mathbf{t}, \mathbf{r}, \mathbf{b}\right) + \lambda_{\mathbf{t}} \rho_{\mathbf{t}}(\mathbf{t}) + \lambda_{\mathbf{r}} \rho_{\mathbf{r}}(\mathbf{r}) + \lambda_{\mathbf{b}} \rho_{\mathbf{b}}(\mathbf{b}) \tag{2}$$

where $\lambda_{\mathbf{t}}$, $\lambda_{\mathbf{m}}$ and $\lambda_{\mathbf{b}}$ are hyperparameters controlling the amount of regularisation of the point cloud and background respectively. Following a Bayesian viewpoint, the regularisation terms $\rho_{\mathbf{t}}(\mathbf{t})$, $\rho_{\mathbf{r}}(\mathbf{r})$ and $\rho_{\mathbf{b}}(\mathbf{b})$ can be seen as the negative log-prior distributions of the point cloud and background levels, respectively. Under the following assumptions:

1. only one surface per pixel, which reduces to fixing the total number of points to $N_{\mathbf{\Phi}} = N_{\mathrm{r}} N_{\mathrm{c}}$,

2. negligible background levels or removed by a preprocessing step [1, 8] (also equating to $\rho_{\mathbf{b}}(\mathbf{b}) = 0$),

3. and convex regularisation terms $\rho_{\mathbf{t}}(\mathbf{t})$ and $\rho_{\mathbf{r}}(\mathbf{r})$,

problem (2) is convex and has a unique minimiser and it is usually solved by SPIRAL [9] or ADMM [10], which take into account the non-Lipschitz globality of $\nabla_{\mathbf{r}} g\left(\mathbf{t}, \mathbf{r}, \mathbf{b}\right)$. However, these assumptions can be too restrictive for practical implementation, as they do not allow for a variable number of surfaces per pixel. Moreover, the depth and intensity regularisations are decoupled, which hinders any improvement of the intensity estimates by using depth information.

In the multiple-surface per pixel scenario, the ℓ_1 (referred to as SPISTA) [5] and ℓ_{21}+TV [11] algorithms by-pass the problem related to the unknown number of points by estimating a vectorised data cube of intensities $\mathbf{r} \in \mathbb{R}^{N_\mathrm{r}N_\mathrm{c}T}$, where the active depths $\mathbf{t}$ are implicitly given by the non-zero entries of $\mathbf{r}$. Convex priors are then assigned to $\mathbf{r}$, such that (2) is convex and has a unique minimiser. However, this formulation presents disadvantages:

1. The estimated values $\mathbf{r}$ are generally not sparse enough (over-estimation of the number of points) and the gradient involves a dense computation over the complete cube, implying that these algorithms can come with a high computational complexity.
2. The TV-based regularisation term $\rho_\mathbf{r}(\mathbf{r})$ promotes volumetric smoothness, which generally results in poor reconstruction quality and the need of empirical post-processing steps, as the reconstructed surfaces should be manifolds.

The algorithm presented in ManiPoP [3] proposes a model based on spatial point processes to promote manifolds, which improves the results of ℓ_1 [5] and ℓ_{21}+TV [11]. However, since the reconstruction is performed via a reversible jump MCMC algorithm, the method has a intrinsically sequential structure, which is difficult to be parallelised, making the algorithm not well adapted for real-time processing.

Novel reconstruction algorithm

In this work, we avoid the issues induced by the high dimensionality of the intensity parameters involved in the volumetric formulation [5, 11], while allowing for a variable number of surfaces per pixel. More precisely, here $\mathbf{t}$ and $\mathbf{r}$ are sets of variable size $N_{\mathbf{\Phi}}$.

Reparametrisation In a similar fashion to other optimisation algorithms assuming Poisson observation noise [12, 13], we introduce the transformation

$$m_n = \log r_n \quad \forall n = 1, \ldots, N_{\mathbf{\Phi}} \tag{3}$$

and fix a maximum intensity $m_n \in (-\infty, \log r_{\max}]$. This change of variables and additional constraint ensure that the likelihood remains globally Lipshitz differentiable with respect to $\mathbf{r}$. The vectorised set of log-intensity values is denoted by $\mathbf{m} = [m_1, \ldots, m_{N_\Phi}]^T$. Analogously, we estimate the log-background levels, i.e., $l_{i,j} = \log b_{i,j}$, denoting the vectorised log-background image as $\mathbf{l} = [l_1, \ldots, l_{N_\mathrm{r}N_\mathrm{c}}]^T$. The resulting negative log-likelihood function under this parametrisation is

$$g\left(\mathbf{t}, \mathbf{m}, \mathbf{l}\right) = -\sum_{i=1}^{N_\mathrm{c}} \sum_{j=1}^{N_\mathrm{r}} \sum_{t=1}^{T} z_{i,j,t} \log \left(\sum_{\mathcal{N}_{i,j}} g_{i,j} e^{m_n} h_{i,j}(t - t_n) + g_{i,j} e^{l_{i,j}} \right) + g_{i,j} e^{l_{i,j}} T + \sum_{\mathcal{N}_{i,j}} g_{i,j} e^{m_n} \tag{4}$$

under the assumption of a normalised impulse response, i.e., $\sum_{t=1}^{T} h_{i,j}(t) = 1$ for all the pixels (i, j).

Proximal gradient steps To solve the general problem in (2), we follow the structure of PALM [14]: the proposed algorithm alternates between the optimisation of three blocks of variables ($\mathbf{t}$, $\mathbf{m}$ and $\mathbf{l}$), applying a proximal gradient update on each step, i.e.,

$$\begin{cases} \tilde{\mathbf{t}} & \leftarrow \mathbf{t}^s - \mu_t^s \nabla_{\mathbf{t}} g\left(\mathbf{t}^s, \mathbf{m}^s, \mathbf{l}^s\right) \\ \mathbf{t}^{s+1} & \leftarrow \underset{\mathbf{t}}{\arg\min}\, \lambda_{\mathbf{t}} \rho_{\mathbf{t}}(\mathbf{t}) + \frac{1}{2\mu_t^s} ||\mathbf{t} - \tilde{\mathbf{t}}||_2^2 \end{cases} \tag{5}$$

$$\begin{cases} \tilde{\mathbf{m}} & \leftarrow \mathbf{m}^s - \mu_m^s \nabla_{\mathbf{m}} g\left(\mathbf{t}^{s+1}, \mathbf{m}^s, \mathbf{l}^s\right) \\ \mathbf{m}^{s+1} & \leftarrow \underset{\mathbf{m}}{\arg\min}\, \lambda_{\mathbf{m}} \rho_{\mathbf{m}}(\mathbf{m}) + \frac{1}{2\mu_m^s} ||\mathbf{m} - \tilde{\mathbf{m}}||_2^2 \end{cases} \tag{6}$$

and

$$\begin{cases} \tilde{\mathbf{l}} & \leftarrow \mathbf{l}^s - \mu_l^s \nabla_{\mathbf{l}} g\left(\mathbf{t}^{s+1}, \mathbf{m}^{s+1}, \mathbf{l}^s\right) \\ \mathbf{l}^{s+1} & \leftarrow \underset{\mathbf{l}}{\arg\min}\, \lambda_{\mathbf{l}} \rho_{\mathbf{l}}(\mathbf{l}) + \frac{1}{2\mu_l^s} ||\mathbf{l} - \tilde{\mathbf{l}}||_2^2 \end{cases} \tag{7}$$

where μ_t, μ_m and μ_l are the step sizes for the depths, log-intensity and background levels respectively. The gradients with respect to the depth, log-intensity and background levels are denoted by $[\nabla_{\mathbf{t}} g\left(\mathbf{t}, \mathbf{m}, \mathbf{l}\right)]_n = \partial g\left(\mathbf{t}, \mathbf{m}, \mathbf{l}\right)/\partial t_n$, $[\nabla_{\mathbf{m}} g\left(\mathbf{t}, \mathbf{m}, \mathbf{l}\right)]_n = \partial g\left(\mathbf{t}, \mathbf{m}, \mathbf{l}\right)/\partial m_n$ and $[\nabla_{\mathbf{l}} g\left(\mathbf{t}, \mathbf{m}, \mathbf{l}\right)]_n = \partial g\left(\mathbf{t}, \mathbf{m}, \mathbf{l}\right)/\partial l_n$.

Depth denoising One of the key contributions of this paper is to extend the ideas introduced for plug-and-play denoising [15] to 3D point clouds, replacing the proximal operator of (5) by the APSS algorithm, i.e.,

$$\mathbf{t}^{s+1} \leftarrow \text{APSS}(\tilde{\mathbf{t}}). \tag{8}$$

The APSS algorithm fits a continuous surface to the set of points defined by $\mathbf{t}$, using spheres as local primitives [16, 17]. The algebraic spheres are parametrised by the vector $\mathbf{u} = [u_0, \ldots, u_4]^T$, according to the scalar field $\phi : \mathbb{R}^3 \to \mathbb{R}$, that is

$$\phi_{\mathbf{u}}(\mathbf{c}) = [1, \mathbf{c}^T, \mathbf{c}^T\mathbf{c}]\mathbf{u}. \tag{9}$$

For each 3D point $\mathbf{c}_n = [i, j, t_n]^T$, the local sphere is fitted by minimising the following problem

$$\underset{\mathbf{u}}{\arg\min} \sum_{r=1}^{N_{\mathbf{\Phi}}} w\left(||\mathbf{c}_n - \mathbf{c}_{\mathrm{r}}||_{\Sigma}\right) \phi_{\mathbf{u}}^2(\mathbf{c}_{\mathrm{r}}) \tag{10}$$

where $w(t) = (1 - t^2)^4$ is a smooth compactly supported weight function and $||\mathbf{c}||_{\Sigma} = \mathbf{c}^T\Sigma\mathbf{c}$ is a metric of choice, with Σ a diagonal matrix with positive entries, which controls the degree of low-pass filtering of the surface. In particular, $w(t)$ is chosen with diagonal entries, i.e.,

$$\Sigma = \begin{pmatrix} d_x & 0 & 0 \\ 0 & d_y & 0 \\ 0 & 0 & d_t \end{pmatrix} \tag{11}$$

In all of the experiments, we set $d_x = d_y = 1$, such that only the 8 closest neighbouring pixels have strong weights, and d_t to be the minimum distance between two surfaces in the same transverse pixel, which is chosen according to the bin width of the lidar system to have a physical meaning (see also the experimental analysis conducted in 'Setting the hyperparameters' below). Interestingly, we chose the same distance as the hard constraint between points in the same pixel in ManiPoP [3]. The fitting is performed in real-world coordinates, using the camera mapping $f(\cdot)$. Supplementary Figure 1 illustrates the surface fitting performed by APSS. The implicit definition of the scalar field is evaluated in every pixel with at least 3 neighbours, filling any holes and dilating the existing surfaces. Similarly to the *almost orthogonal* projection [18], we repeat the fitting process until there is no significant change in the projected point.

Intensity denoising The proximal operator of the log-intensity update in (6) is replaced by a denoising step using the manifold metrics. In this work, we simply consider a low-pass filter using the nearest neighbours of each point, as in ISOMAP [19]: each log-intensity m_n is updated as

$$m_n^{s+1} = \beta m_n^s + (1-\beta) \sum_{n' \in \mathcal{M}(m_n^s)} \frac{m_{n'}^s}{\# \mathcal{M}(m_n^s)} \tag{12}$$

where β is a coefficient controlling the amount of filtering, $\mathcal{M}(m_n)$ is the set of spatial neighbours m_n and $\# \mathcal{M}(m_n)$ denotes the total number of neighbours. Hence, the proximal step is summarised as

$$\mathbf{m}^{s+1} \leftarrow \text{Manifold denoising}(\tilde{\mathbf{m}}) \tag{13}$$

More elaborate filters could also be applied, using the manifold metrics defined by the implicit mean least squares surface [20]. After the denoising step, we remove the points with intensity r_n lower than a given threshold r_{min}. This step prevents the algorithm from growing surfaces without bounds.

Background denoising The proximal operator used for $\mathbf{l}$ depends on the prior assumptions that can be made about the spatial configuration of the spurious detections. In bistatic raster-scanning systems [5], background counts are not necessarily spatially correlated, thus the proximity operator can be chosen as the identity operator. In monostatic raster-scanning systems [21] or lidar arrays (e.g., the Princeton Lightwave lidar used in this paper), the background detections appear as a passive image of the imaged scene. Thus, a spatial regularisation is useful to improve the background estimates. In this case, we use a Gaussian Markov random field regularisation [22], i.e., $\rho_{\mathbf{l}}(\mathbf{l}) = \mathbf{l}^T \mathbf{P} \mathbf{l}/2$, where $\mathbf{P}$ is the Laplacian 2D filter. The proximal operator is thus

$$\mathbf{l}^{s+1} \leftarrow (\mathbf{I} + \lambda_{\mathbf{l}} \mu_l^s \mathbf{P})^{-1} \tilde{\mathbf{l}} \tag{14}$$

where $\mathbf{I}$ is the identity matrix. This denoising step can be quickly computed using the fast Fourier transform (FFT). The proximal operator can also be replaced by an off-the-shelf image denoising algorithm, such as NLM [23] or BM3D [24], at the cost of a higher computational load.

Supplementary Note 2: Setting the parameters

Step sizes Assuming the number of points is constant, the step sizes at iteration s should verify $\mu_t^s < \frac{1}{L_t^s}$, $\mu_m^s < \frac{1}{L_m^s}$ and $\mu_l^s < \frac{1}{L_l^s}$, where L_t^s, L_m^s and L_l^s are the Lipschitz constants of $\nabla_{\mathbf{t}} g\left(\mathbf{t}^s, \mathbf{m}^s, \mathbf{l}^s\right)$, $\nabla_{\mathbf{m}} g\left(\mathbf{t}^s, \mathbf{m}^s, \mathbf{l}^s\right)$ and $\nabla_{\mathbf{l}} g\left(\mathbf{t}^s, \mathbf{m}^s, \mathbf{l}^s\right)$ respectively [25, 14]. The value of L_t^s can be approximated by assuming that the non-diagonal entries of the Hessian matrix, $\partial g\left(\mathbf{t}, \mathbf{m}^s, \mathbf{l}^s\right)/\partial m_n \partial m_k$ with $k \neq n$, are negligible. Under this approximation, the Lipschitz constant is

$$L_t^s = \max\left\{ \frac{\partial^2 g\left(\mathbf{t}, \mathbf{m}^s, \mathbf{l}^s\right)}{\partial t_n^2} \quad n = 1, \dots, N_{\mathbf{\Phi}} \right\}. \tag{15}$$

If the impulse response has a Gaussian shape, i.e., $h_{i,j}(t) \propto \exp(-(t/\sigma)^2/2)$, the partial derivatives can be computed analytically, leading to

$$L_t^s \leq \frac{1}{\sigma^2} \max_{i,j} \sum_{t=1}^{T} z_{i,j,t} \tag{16}$$

which only depends on the width of the impulse response and the maximum number of photons per pixel. Thus, we set a fixed step size $\mu_t^s = \mu_t$, dropping the dependence on the iteration s. The values of L_m^s and L_l^s are bounded by the maximum point intensity and background level, that is

$$L_m^s \leq \sum_{t=1}^{T} h_{i,j}(t) \max_n e^{m_n^s} \tag{17}$$

$$L_l^s \leq T \max_{i,j} e^{l_{i,j}}. \tag{18}$$

We bound the maximum intensity, such that L_m^s is upper bounded irrespective of the iteration s. The value of μ_l^s is set to $1/(T \max_{i,j} e^{l_{i,j}^s})$, according to the maximum background level at each iteration.

The aforementioned rules for setting step sizes only guarantee the convergence to a local minimum if the dimension of the problem remains fixed [14]. The overall problem is highly non-convex and changes dimension at each step. Thus, the outcome of the algorithm depends on the initialisation. However, as shown in Supplementary Note 3, the algorithm converges to fixed points in practical scenarios and is robust to different initialisations.

Hyperparameters Supplementary Figures 2 to 5 illustrate the impact of the hyperparameters on the reconstruction performance using the "polystyrene head with backplane" and "polystyrene head without backplane" datasets, with the aim of providing basic guidelines to select them.

Supplementary Note 3: Initialisation

The initialisation step is designed to provide a coarse estimate, while being fast and easily parallelisable. If at most one surface per pixel is expected, then the classical cross-correlation can be applied. Supplementary Figure 11 shows the initialisation (top row) and achieved reconstructions (bottom row) for different decimations of the cross-correlation initialisation (i.e., the output of the cross-correlation is decimated before finding the bin that realises the maximum). Decimating the cross-correlation function reduces to considering a reduced number of admissible ranges, which in turn reduces the computational complexity of the initialisation. For instance, Supplementary Fig. 11 (b) uses only three admissible ranges (top subplot). Yet, the algorithm yields the same reconstruction even if only 0.11% of the total cross-correlation is computed. As shown in Supplementary Fig. 12, the algorithm recovers the same amount of true points for a wide range of initialisation, converging to the same likelihood value. This approach can be used to further accelerate the algorithm.

In a general setting where multiple surfaces may be present, we initialise the algorithm with a multi-surface extension of the classic cross-correlation. We propose two different alternatives depending on the sparsity of the recorded histograms:

- Lidar arrays present dense histograms, such that we can use the Anscombe transform [26] to stabilise the variance of the Poisson noise. After the transform, the matching pursuit algorithm [27] is used to find the M most prominent surfaces on each pixel, as summarised in Algorithm 1. The parameter M is user defined and in the experiments presented here, we chose $M = 3$.

Algorithm 1 Dense VST-MP initialisation

Input: Lidar waveforms $\mathbf{Z}$, maximum number of surfaces per pixel M, $\mathbf{z}_{i,j} = [z_{i,j,1}, \dots, z_{i,j,T}]^T$
Main loop: Process each pixel (i, j) in parallel
$\tilde{\mathbf{z}}_{i,j} \leftarrow \mathrm{VST}(\mathbf{z}_{i,j})$
$t_1, \dots, t_M \leftarrow$ Matched Pursuit using $\tilde{\mathbf{z}}_{i,j}$ and atoms given by the shifted impulse response $h_{i,j}(t)$
for $s = 1, \dots, M$ **do**
  $m_s \leftarrow \log(\sum_{t:h_{i,j}(t-t_s)\neq 0} z_{i,j,t})$
end for
$l_{i,j} = \log(\sum_{t\in\mathcal{T}} z_{i,j,t} / \sum_{t\in\mathcal{T}} 1)$ where $\mathcal{T} = \{t : h_{i,j}(t - t_s) \neq 0 \quad \forall s = 1, \dots, M\}$
Output: Initial estimates $(\mathbf{t}^0, \mathbf{m}^0, \mathbf{l}^0)$

- Histograms collected using single-photon lidar systems with high temporal resolution ($<$ 20ps), e.g., raster-scanning systems, generally present a large number of sparsely populated bins, hindering any dense computations using the Anscombe transform. In this case, we find the M most prominent peaks by iteratively using the cross-correlation estimate and removing the photons associated with the peak, as shown in Algorithm 2.

Algorithm 2 Sparse MP initialisation

1: **Input:** Lidar waveforms $\mathbf{Z}$, maximum number of surfaces per pixel M, $\mathbf{z}_{i,j} = [z_{i,j,1}, \ldots, z_{i,j,T}]^T$
2: **Main loop:** Process each pixel (i, j) in parallel
3: **for** $s = 1, \ldots, M$ **do**
4: $\quad t_s \leftarrow$ Cross-correlation maximum$(\mathbf{z}_{i,j})$
5: $\quad m_s \leftarrow \log(\sum_{t:h_{i,j}(t-t_s)\neq 0} z_{i,j,t})$
6: $\quad z_{i,j,t} \leftarrow 0 \quad \forall t : h_{i,j}(t - t_s) \neq 0.$
7: **end for**
8: $l_{i,j} = \log(\sum_{\mathcal{T}} z_{i,j,t} / \sum_{\mathcal{T}} 1)$ where $\mathcal{T} = \{t : h_{i,j}(t - t_s) \neq 0 \quad \forall s = 1, \ldots, M\}$
9: **Output:** Initial estimates $(\mathbf{t}^0, \mathbf{m}^0, \mathbf{l}^0)$

Supplementary Note 4: Parallel implementation

Pseudo-code of the full algorithm is presented in Algorithm 3. Our implementation runs completely on a GPU, only exchanging the lidar waveforms and final output with the CPU. The parallel structures of the initialisation and main algorithm allow for efficient GPU implementation, as each parallel thread only requires the information of a local subset of photon measurements and 3D points.

As the initialisation algorithms process every pixel independently, one parallel thread is executed per lidar pixel. The general per-pixel complexity of the dense case is $\mathcal{O}(MT \log T)$, whereas the complexity of the sparse algorithm is $\mathcal{O}(Mk)$, where k is the number of bins with one or more photons.

The gradient and denoising steps of the main algorithm have different parallel implementations. Each of the parallel threads processes one lidar waveform in the gradient steps of (5) and (6), as they can be processed independently of the rest due to the separable structure of the negative log-likelihood. The per-pixel complexity for the depth and log-intensity gradients is $\mathcal{O}(k)$ with k the number of non-zero bins in the compact support of the impulse response centred in the existing points, which is smaller than $\mathcal{O}(T \log T)$ needed for algorithms working on a dense intensity cube such as SPISTA [5] or ℓ_{21}+TV [11], especially when the number of histogram bins T is large. The background gradient step in (6) has a complexity of $\mathcal{O}(k)$, where k is the number of active photon pixels in the processed histogram. Both the APSS and intensity denoising steps run one thread per world-coordinates pixels, making use of the shared GPU memory (a gather operation [28]) to efficiently read the information of its neighbours. The main bottleneck of these steps is given by the memory reads during the gather operation, which can be reduced by considering fewer neighbours at the cost of a potentially degraded reconstruction. Note that the proposed method has minimal memory requirements. In contrast to convex alternatives [5, 7], which require the storage of a dense 3D cube of intensity estimates of size $\mathcal{O}(N_\mathrm{r} N_\mathrm{c} T)$, the proposed method only stores the estimated point cloud of size $\mathcal{O}(N_\mathrm{r} N_\mathrm{c})$.

The complexity of the algorithm is generally dominated by the gradient steps, which depend on the number of photons (active bins) per pixel. For example, the proposed method might run faster on a large array with few photon detections than a smaller array with densely populated histograms. To illustrate this, consider the execution times of the large raster-scan dataset (13 ms) and the Princeton Lightwave dataset (20 ms). While being significantly smaller, the 32×32 array has dense histograms of 153 bins with non-

zero counts. On the other hand, the 141×141 raster scan dataset has a mean photon count of 3 photons per pixel, hence having approximately 3 active bins per pixel. The effective data size in the former case is $32 \times 32 \times 153 = 156672$, whereas in the latter is $141 \times 141 \times 3 \times 2 = 119286$ (where the last term in the multiplication is due to the bin number indicator in a sparse representation). The latter data size is smaller than the 32×32 array, hence the faster processing. Moreover, as the algorithm's complexity is driven by the amount of computation within a pixel, it is more intensive to process 153 bins than 4 active bins.

Algorithm 3 Real-time single-photon 3D imaging (RT3D)

Input: Lidar waveforms $\mathbf{Z}$ and camera parameters $f(\cdot)$
Initialisation:
$s \leftarrow 0$
$(\mathbf{t}^0, \mathbf{m}^0, \mathbf{l}^0) \leftarrow$ Algorithm 1 (array) or Algorithm 2 (raster-scan)
Main loop:
while $s < N_i$ **do**
  $\mathbf{t}^{s+1} \leftarrow$ Point cloud denoising $(\mathbf{t}^s - \mu_t \nabla_{\mathbf{t}} g(\mathbf{t}^s, \mathbf{m}^s, \mathbf{l}^s))$
  $\mathbf{m}^{s+1} \leftarrow$ Manifold denoising $(\mathbf{m}^s - \mu_m \nabla_{\mathbf{m}} g(\mathbf{t}^{s+1}, \mathbf{m}^s, \mathbf{l}^s))$
  $\mathbf{l}^{s+1} \leftarrow \mathbf{l}^s - \mu_l^s \nabla_{\mathbf{l}} g(\mathbf{t}^{s+1}, \mathbf{m}^{s+1}, \mathbf{l}^s)$
  if the lidar system is raster-scan monostatic or array **then**
    $\mathbf{l}^{s+1} \leftarrow$ Image denoising $(\mathbf{l}^{s+1})$
  end if
  $s \leftarrow s + 1$
end while
Output: Final estimates $(\mathbf{t}^{N_i}, \mathbf{m}^{N_i}, \mathbf{l}^{N_i})$

Supplementary Note 5: Operation boundary conditions

We generated 100 synthetic lidar cubes for SBR values in $[0.01, 100]$ and mean photons per pixels in $[0.1, 100]$ using the ground truth point cloud, data cube size and impulse response from the "polystyrene head without backplane" dataset. Supplementary Figure 10 shows a comparison of the proposed method with the standard cross-correlation algorithm. To account for pixels without objects, we post-processed the output of cross-correlation by removing points below a normalised intensity of 10%. The proposed method performs well in a wider range of conditions, achieving reconstructions with ≈ 0.1 photons per pixel and up to signal-to-noise background ratio of 0.01 (with 100 photons per pixel or more). Moreover, cross-correlation generates many orders of magnitude more false detections than the new method. Interestingly, the proposed algorithm exhibits a sharper transition in the detection of true points, meaning that, for a given signal-to-background ratio, either none or most of the points will be found depending on the recorded photon count. The novel method achieves smaller depth and intensity absolute errors than cross-correlation in all conditions, as it exploits the manifold structure of the scene. Moreover, the proposed algorithm also achieves a significantly smaller background NMSE, capturing the spatial correlation in the background image.

Supplementary Note 6: Beyond the APSS denoiser

In this work, we focus on the APSS denoiser to target real-time performance, profiting from the parallel structure and closed-form updates. However, we could imagine other choices with different trade-offs between execution time, memory requirement and reconstruction quality [29]. For example, a straightforward alternative is the simple point set surfaces (SPSS) denoiser instead of APSS. The proposed method provides a framework to incorporate different types of prior information, avoiding the need to develop specific algorithms for single-photon lidar. APSS only relies on a local surface smoothness prior, whereas more sophisticated denoisers exploit more complex prior knowledge on the point cloud structure [30]. Non-local correlations between point cloud patches using a dictionary learning approach [31]. Higher-level knowledge on the scene, such as the presence of buildings or humans could be also exploited through dedicated denoisers. For instance, an adaptive partitioning algorithm [32] uses planes to denoise point clouds of building facades, being adapted for remote sensing/outdoor applications. Finally, we could also profit from available 3D data using data-driven denoisers. In this direction, we can use algorithms that fit templates of possible objects [33] or profit from recent advances in graph convolutional neural networks [34], which are especially designed to handle point cloud structures [35, 36].

Supplementary Note 7: Comparison with state-of-the-art reconstruction algorithms

We evaluated the proposed method using 4 lidar datasets acquired with different systems, summarised in Supplementary Table 1. The "polystyrene head with backplane" dataset, shown in the main paper, corresponds to the classical setting with one surface in almost all pixels. The "polystyrene head without backplane" dataset, shown in Supplementary Fig. 6, contains at most one surface per pixel. The "human behind camouflage netting" and "mannequin behind a scattering object", shown in Supplementary Fig. 7, have multiple surfaces per pixel. We compare our results to those obtained with standard cross-correlation, a state-of-the-art single-surface algorithm [1], three multiple-surface reconstruction algorithms SPISTA [5], ℓ_{21}+TV [7] and ManiPoP [3], and a target detection algorithm [6]. Supplementary Figures 6 and 7 show the 3D reconstructions obtained by the competing algorithms for each dataset, whereas their execution time are presented in Supplementary Table 2. Supplementary Figure 8 shows the percentage of true detections and number of false detections as a function of the maximum distance between a ground truth point and an estimated point.

Supplementary Methods

3D lidar array

A schematic diagram of the lidar system, which was based on the 32×32 single-photon array Kestrel camera produced by Princeton Lightwave, is shown in Supplementary Fig. 13. The system was implemented as a

bistatic arrangement - the illuminating transmit (laser) channel and the collecting receive (camera) channel were not co-axial - with the centres of the apertures separated by about 125 mm. This configuration was used in order to avoid potential issues that could arise in a co-axial (monostatic) system due to back reflections from the optical components causing damage to the sensitive focal plane array of the Kestrel camera. The bistatic optical configuration meant that a slight re-alignment of the illumination channel, relative to the receive (camera) channel, was required for scenes at different distances from the system. Both the camera and laser were mounted on a single breadboard with the optical setup for the illumination channel mounted on a stage which enabled controlled adjustments to be made to the pitch and yaw of the illuminating beam, so that it could be positioned accurately relative to the field of view of the camera. Another camera (Ninox 640 VIS-SWIR, from Raptor Photonics) was also mounted on the breadboard and used to help align the system to the scene of interest.

The operating wavelength for the system was chosen as 1550 nm - this wavelength corresponds to a high transmission window in the atmosphere, with the unwanted contribution from solar background being significantly lower when compared to shorter wavelengths, and it is eye-safe at significantly higher power levels than for wavelengths in the retinal hazard region of the spectrum (which extends from 400 to 1400 nm). The BKtel fibre laser (HFL-240am series) had a central wavelength of 1550 nm and the pulse width was measured to be 413 ps at the operating parameters used in these measurements. It was run at a repetition rate of 150.421 kHz (this clock signal was provided by the Kestrel camera), and the resulting average optical output power was approximately 220 mW (for a laser drive current of 3 A). A neutral density (ND) filter with an optical density of 0.5 and transmission of approximately 32% at $\lambda = 1550$ nm was used to reduce the average optical power level to approximately 70 mW to avoid saturating the sensitive detector. The output fibre from the laser module was connected to a reflective collimation package and the exiting beam was then passed through a 12 nm FWHM bandpass filter with a centre wavelength of 1550 nm in order to remove any amplified spontaneous emission that was present. A beam expander arrangement consisting of a pair of lenses (with effective focal lengths of approximately 10 mm and 75 mm) housed in a zoom mechanism enabled the diameter of the illuminating beam at the scene of interest to be adjusted to match the field of view of the camera.

The Kestrel camera had an InGaAs/InP SPAD detector array and at the operating wavelength of 1550 nm, the elements in the array had a quoted photon detection efficiency of approximately 25% and a measured dark count rate of approximately 320 kcps. The camera was operated in time-of-flight mode and configured to operate with 250 ps timing bins, a gate duration of 40 ns, which corresponds to a total of 160 histogram bins, and was equivalent to a measurement depth range of 6 metres. The camera was operated at a frame rate of 150.421 kHz (this was close to the expected maximum frame rate of the camera). In order to acquire an accurate instrumental response of the system (i.e., accurate estimations of $h_{i,j}(t)$ and $g_{i,j}$) a long-acquisition measurement of a uniform, cooperative surface (Spectralon, Labsphere, Inc) was made in dark laboratory conditions over a short stand-off distance of 2 metres. Due to a small amount of latency present in the timing electronics of each SPAD detector, the instrumental response of each pixel in the camera array is non-identical. This is taken in to consideration during data reconstruction by using a separate instrumental response for each pixel.

A 500 mm effective focal length lens operating at f/7 (manufactured by Optec, and designed for use in the 900 to 1700 nm wavelength region) was attached to the camera to collect the scattered return photons

from the scene. This resulted in a field of view of approximately 2×2 metres at the standoff distance of 320 metres, i.e., each individual pixel covered an area of approximately 65×65 mm. In order to minimise the amount of background light detected, a pair of high performance passive spectral filters was mounted between the rear element of the lens and the sensor of the camera - one was a longpass filter with a cut-on wavelength of 1500 nm, and the other was a 9 nm full width half maximum (FWHM) bandpass filter with a centre wavelength of 1550 nm.

Supplementary References

[1] Rapp, J. & Goyal, V. K. A few photons among many: Unmixing signal and noise for photon-efficient active imaging. *IEEE Trans. Comput. Imag.* **3**, 445–459 (2017).

[2] Altmann, Y., Ren, X., McCarthy, A., Buller, G. S. & McLaughlin, S. Lidar waveform-based analysis of depth images constructed using sparse single-photon data. *IEEE Trans. Image Process.* **25**, 1935–1946 (2016).

[3] Tachella, J. *et al.* Bayesian 3D reconstruction of complex scenes from single-photon lidar data. *SIAM Journal on Imaging Sciences* **12**, 521–550 (2019).

[4] Tobin, R. *et al.* Long-range depth profiling of camouflaged targets using single-photon detection. *Opt. Eng.* **57**, 1 – 10 (2017).

[5] Shin, D., Xu, F., Wong, F. N., Shapiro, J. H. & Goyal, V. K. Computational multi-depth single-photon imaging. *Opt. Express* **24**, 1873–1888 (2016).

[6] Altmann, Y., Ren, X., McCarthy, A., Buller, G. S. & McLaughlin, S. Robust Bayesian target detection algorithm for depth imaging from sparse single-photon data. *IEEE Trans. Comput. Imag.* **2**, 456–467 (2016).

[7] Halimi, A., Tobin, R., McCarthy, A., McLaughlin, S. & Buller, G. S. Restoration of multilayered single-photon 3D lidar images. In *Proc. 25th European Signal Processing Conference (EUSIPCO)*, 708–712 (Kos Island, Greece, 2017).

[8] Shin, D., Kirmani, A., Goyal, V. K. & Shapiro, J. H. Photon-efficient computational 3-D and reflectivity imaging with single-photon detectors. *IEEE Trans. Comput. Imag.* **1**, 112–125 (2015).

[9] Harmany, Z. T., Marcia, R. F. & Willett, R. M. This is SPIRAL-TAP: Sparse poisson intensity reconstruction algorithms: Theory and practice. *IEEE Trans. Image Process.* **21**, 1084–1096 (2012).

[10] Figueiredo, M. A. T. & Bioucas-Dias, J. M. Restoration of Poissonian images using alternating direction optimization. *IEEE Trans. Image Process.* **19**, 3133–3145 (2010).

[11] Halimi, A. *et al.* Restoration of intensity and depth images constructed using sparse single-photon data. In *Proc. 24th European Signal Processing Conference (EUSIPCO)*, 86–90 (Budapest, Hungary, 2016).

[12] Salmon, J., Harmany, Z., Deledalle, C.-A. & Willett, R. Poisson noise reduction with non-local PCA. *Journal of Mathematical Imaging and Vision* **48**, 279–294 (2014).

[13] Marais, W. & Willett, R. Proximal-gradient methods for Poisson image reconstruction with BM3D-based regularization. In *Proc. 7th International Workshop on Computational Advances in Multi-Sensor Adaptive Processing (CAMSAP)*, 1–5 (Curacao, Dutch Antilles, 2017).

[14] Bolte, J., Sabach, S. & Teboulle, M. Proximal alternating linearized minimization for nonconvex and nonsmooth problems. *Mathematical Programming* **146**, 459–494 (2014).

[15] Sreehari, S. *et al.* Plug-and-play priors for bright field electron tomography and sparse interpolation. *IEEE Trans. Comput. Imag.* **2**, 408–423 (2016).

[16] Guennebaud, G. & Gross, M. Algebraic point set surfaces. *ACM Trans. Graph.* **26** (2007).

[17] Guennebaud, G., Germann, M. & Gross, M. Dynamic sampling and rendering of algebraic point set surfaces. *Computer Graphics Forum* **27**, 653–662 (2008).

[18] Alexa, M. & Adamson, A. On normals and projection operators for surfaces defined by point sets. In *Proc. Eurographics Conference on Point-Based Graphics*, 149–155 (Aire-la-Ville, Switzerland, 2004).

[19] Tenenbaum, J. B., Silva, V. d. & Langford, J. C. A global geometric framework for nonlinear dimensionality reduction. *Science* **290**, 2319–2323 (2000).

[20] Liang, J. & Zhao, H. Solving partial differential equations on point clouds. *SIAM Journal on Scientific Computing* **35**, A1461–A1486 (2013).

[21] Pawlikowska, A. M., Halimi, A., Lamb, R. A. & Buller, G. S. Single-photon three-dimensional imaging at up to 10 kilometers range. *Opt. Express* **25**, 11919–11931 (2017).

[22] Rue, H. & Held, L. *Gaussian Markov random fields: theory and applications* (CRC press, 2005).

[23] Buades, A., Coll, B. & Morel, J. . A non-local algorithm for image denoising. In *Proc. Computer Society Conference on Computer Vision and Pattern Recognition (CVPR'05)*, vol. 2, 60–65 (San Diego, USA, 2005).

[24] Dabov, K., Foi, A., Katkovnik, V. & Egiazarian, K. Image denoising by sparse 3-D transform-domain collaborative filtering. *IEEE Trans. Image Process.* **16**, 2080–2095 (2007).

[25] Parikh, N. & Boyd, S. Proximal algorithms. *Foundations and Trends in Optimization* **1**, 127–239 (2014).

[26] Anscombe, F. J. The transformation of Poisson, binomial and negative-binomial data. *Biometrika* **35**, 246–254 (1948).

[27] Mallat, S. G. & Zhang, Z. Matching pursuits with time-frequency dictionaries. *IEEE Transactions on Signal Processing* **41**, 3397–3415 (1993).

[28] Sanders, J. & Kandrot, E. *CUDA by example: An introduction to general-purpose GPU programming* (Addison-Wesley Professional, 2010).

[29] Chandrasekaran, V. & Jordan, M. I. Computational and statistical tradeoffs via convex relaxation. *In Proc. of the National Academy of Sciences* **110**, 1181–1190 (2013).

[30] Berger, M. *et al.* A survey of surface reconstruction from point clouds. *Computer Graphics Forum* **36**, 301–329 (2017).

[31] Xiong, S., Zhang, J., Zheng, J., Cai, J. & Liu, L. Robust surface reconstruction via dictionary learning. *ACM Trans. Graph.* **33**, 201:1–201:12 (2014).

[32] Shen, C.-H., Huang, S.-S., Fu, H. & Hu, S.-M. Adaptive partitioning of urban facades. *ACM Trans. Graph.* **30**, 184:1–184:10 (2011).

[33] Nan, L., Xie, K. & Sharf, A. A search-classify approach for cluttered indoor scene understanding. *ACM Trans. Graph.* **31**, 137:1–137:10 (2012).

[34] Bronstein, M. M., Bruna, J., LeCun, Y., Szlam, A. & Vandergheynst, P. Geometric deep learning: Going beyond Euclidean data. *IEEE Signal Processing Magazine* **34**, 18–42 (2017).

[35] Li, Y. *et al.* PointCNN: Convolution on X-transformed points. In *Advances in Neural Information Processing Systems (NIPS 2018)*, 820–830 (Montreal, Canada, 2018).

[36] Wang, Y. *et al.* Dynamic graph CNN for learning on point clouds. *Preprint at https://arxiv.org/abs/1801.07829* (2018).
