# Supplementary material for: Real-time 3D reconstruction from single-photon lidar data using plug-and-play point cloud denoisers
Source: Nat Commun. 2019 Nov 1;10:4984. doi: 10.1038/s41467-019-12943-7 (PMC6825222; doi:10.1038/s41467-019-12943-7)
Supplement: Supplementary file 3 — Description of Supplementary Information [file 41467_2019_12943_MOESM3_ESM.pdf]

Description of Additional Supplementary Files

Supplementary Movie 1

Description: 3D videos from lidar array data. The videos were acquired at a stand-off distance of 320 metres from the lidar system in broad daylight. The proposed algorithm is able to process 50 frames per second. We show the reconstructions in different settings: multiple surfaces per pixel, exactly one surface per pixel and at most one surface per pixel.
